# Strengthening Transitions in Care for Patients with ST-Elevation Myocardial Infarction: A Theory-Based Qualitative Study

**DOI:** 10.1016/j.cjco.2025.12.004

**Published:** 2025-12-23

**Authors:** Jacob Crawshaw, Marija Corovic, Muhammad Ajlan, Karen D. Mosleh, Madhu Natarajan, J.D. Schwalm

**Affiliations:** a Centre for Evidence-Based Implementation, Hamilton Health Sciences, Hamilton, Ontario, Canada; b Department of Medicine, McMaster University, Hamilton, Ontario, Canada; c Methodological and Implementation Research Program, Ottawa Hospital Research Institute, Ottawa, Ontario, Canada; d Population Health Research Institute, McMaster University, Hamilton, Ontario, Canada

**Keywords:** transitional care, care coordination, patient engagement, multidisciplinary teams, continuity of care, implementation science

## Abstract

**Background:**

Despite strong evidence for secondary prevention after ST-elevation myocardial infarction (STEMI), adherence to pharmacotherapy and participation in cardiac rehabilitation remain suboptimal. Fragmented transitions from hospital to outpatient care contribute to early discontinuation, inadequate self-management support, and delayed functional and psychological recovery, particularly in regional systems without standardized follow-up care. This study examined barriers and enablers to post-STEMI transitions in care using a theory-informed qualitative approach.

**Methods:**

Semi-structured interviews were conducted with STEMI patients (n = 14), healthcare providers (n = 8), and system leaders (n = 4) within a regional cardiac network in Ontario, Canada. Interview guides were informed by the Theoretical Domains Framework (TDF) and the Consolidated Framework for Implementation Research (CFIR). Data were analyzed using directed content analysis.

**Results:**

Patients reported barriers including knowledge gaps about symptoms and treatment (TDF domain: knowledge), difficulty sustaining behavioural routines (TDF: behavioural regulation domain), and logistical challenges in accessing services (TDF: environmental context and resources domain). Providers and leaders emphasized poor communication across settings (CFIR: networks and communication construct), limited follow-up planning (CFIR: planning construct), and lack of sustainable funding models (CFIR: available resources construct). Enablers included strong social support (TDF: social influences domain), expanded roles for nurse practitioners and pharmacists (TDF: social/professional role and identity domain), and openness to virtual follow-up models (CFIR: adaptability construct). Suggested solutions include structured discharge education, interdisciplinary collaboration, standardized follow-up systems, and fostering a culture supportive of implementation.

**Conclusions:**

Applying behavioural and implementation frameworks identified multilevel barriers and enablers to post-STEMI follow-up. Actionable strategies, such as structured education, interdisciplinary care, and expanded nonphysician roles, could strengthen secondary prevention and improve outcomes.

## Résumé

**Contexte:**

Malgré des preuves solides en faveur de la prévention secondaire après un infarctus du myocarde avec élévation du segment ST (STEMI), l'observance du traitement pharmacologique et la participation à la réadaptation cardiaque restent insuffisantes. La fragmentation des transitions entre l'hôpital et les soins ambulatoires contribue à l'arrêt prématuré du traitement, à un soutien inadéquat à l'autogestion et à un rétablissement fonctionnel et psychologique retardé, en particulier dans les réseaux régionaux dépourvus de suivi standardisé. Cette étude a examiné les obstacles et les facteurs favorables aux transitions post-STEMI dans les soins à l'aide d'une approche qualitative fondée sur la théorie.

**Méthodologie:**

Des entretiens semi-structurés ont été menés auprès de patients ayant subi un STEMI (n = 14), de prestataires de soins de santé (n = 8) et de responsables du système (n = 4) au sein d'un réseau régional en cardiologie (Ontario, Canada). Les guides d'entretien s'appuyaient sur le cadre des domaines théorique (CDT) et le cadre consolidé pour la recherche sur la mise en œuvre (CRMO). Les données ont été analysées à l'aide d'une analyse de contenu dirigée.

**Résultats:**

Les patients ont signalé des obstacles, notamment des lacunes dans leurs connaissances sur les symptômes et le traitement (CDT: Connaissances), des difficultés à maintenir des routines comportementales (CDT: Régulation comportementale) et des défis logistiques pour accéder aux services (CDT: Contexte environnemental et ressources). Les prestataires et les responsables ont souligné la mauvaise communication entre les différents établissements (CRMO: Réseaux et communication), la planification limitée du suivi (CRMO: Planification) et l'absence de modèles de financement durables (CRMO: Ressources disponibles). Les facteurs favorables comprenaient un soutien social solide (CDT: Influences sociales), l'élargissement des rôles des infirmières praticiennes et des pharmaciens (CDT: Rôle et identité sociaux/professionnels) et l'ouverture aux modèles de suivi virtuel (CRMO: Adaptabilité). Les solutions suggérées comprenaient un programme d'éducation structurée à la sortie de l'hôpital, une collaboration interdisciplinaire, des systèmes de suivi standardisés et la promotion d'une culture favorable à la mise en œuvre.

**Conclusions:**

L'application de cadres comportementaux et de mise en œuvre a permis d'identifier des obstacles et des facteurs facilitateurs à plusieurs niveaux pour le suivi post- STEMI. Des stratégies concrètes telles qu'une éducation structurée, des soins interdisciplinaires et l'élargissement des rôles des non-médecins pourraient renforcer la prévention secondaire et améliorer les résultats cliniques.

ST-elevation myocardial infarction (STEMI) is a time-sensitive cardiovascular emergency and remains a leading cause of morbidity and mortality in Canada^1^ and worldwide.^2^^,^^3^ Advances in reperfusion strategies and acute care systems have substantially improved survival in the early phase of STEMI care.^4^ However, long-term outcomes remain suboptimal, largely due to challenges in both secondary prevention and sustained adherence to guideline-directed therapies.^5^, ^6^, ^7^ Despite strong evidence that antiplatelet therapy, statins, beta-blockers, and renin–angiotensin system inhibitors reduce recurrent events and improve survival, up to 50% of patients discontinue at least one of these therapies within the first year after hospital discharge.^8^^,^^9^ In addition, despite its proven benefits, cardiac rehabilitation is underutilized in Canada, with less than one third of eligible patients engaging in these programs.^10^^,^^11^ Together, these gaps underscore the critical importance of improving continuity of care and secondary prevention following STEMI.^12^

Fragmentation in follow-up care after hospital discharge is a major contributor to these suboptimal outcomes. Patients with STEMI often experience discontinuity as they transition from highly protocolized, team-based acute care to more variable and less coordinated outpatient management.^13^^,^^14^ In Canada, follow-up processes have substantial heterogeneity; some patients receive specialized cardiology follow-up, whereas others are managed solely in primary care settings.^10^ This inconsistency can result in medication errors, inadequate support for self-management, and missed opportunities to reinforce the importance of medication adherence and lifestyle changes.^15^ Structural limitations, including shortages in cardiac rehabilitation programs, geographic barriers, and inconsistent access to specialist care, further exacerbate inequities.^16^, ^17^, ^18^ From a patient perspective, the transition can be overwhelming (both functionally and psychologically), characterized by uncertainty, difficulty navigating the system, and challenges in maintaining lifestyle modifications without consistent, structured support.^13^

The Canadian Cardiovascular Society and other international bodies have called for greater emphasis on secondary prevention and longitudinal follow-up for patients with acute coronary syndromes such as STEMI.^19^, ^20^, ^21^ Existing follow-up practices within health systems often lack system integration, multidisciplinary collaboration, and standardized protocols,^22^^,^^23^ and few are informed by theory-driven methods that explicitly address patient-, provider-, and system-level determinants of behaviour.

To address these knowledge gaps, we conducted a theory-based qualitative study to identify barriers and enablers to effective follow-up care post-STEMI and generate insights that can inform the design of standardized, evidence-based, and scalable follow-up models for patients with STEMI.

## Methods

### Study design

We conducted a qualitative study using semi-structured interviews to examine factors that hinder or facilitate effective transitions in care following STEMI.

### Guiding theory

Incorporating implementation science frameworks into cardiovascular care offers an opportunity to systematically identify barriers, tailor interventions, and develop scalable models of care.^24^^,^^25^ In particular, frameworks such as the Theoretical Domains Framework (TDF)^26^^,^^27^ and the Consolidated Framework for Implementation Research (CFIR)^28^ provide structured approaches to identify multilevel determinants of care, offering practical insights into how patient-, provider-, and system-level barriers can be addressed. The TDF synthesizes constructs from multiple behaviour change theories across 14 domains, supporting identification of cognitive, social, and environmental influences on health behaviours. The CFIR provides a structured approach to evaluating multilevel factors influencing the uptake of interventions in healthcare, spanning the following 5 domains: intervention characteristics; inner setting; outer setting; characteristics of individuals; and implementation processes. Combining these frameworks enabled a comprehensive examination of individual, organizational, and system-level influences on post-STEMI care transitions.

### Setting and participants

The study was conducted within a regional cardiac network in Ontario, Canada, which includes acute academic centres and affiliated community hospitals that provide coordinated STEMI care, including reperfusion, secondary prevention, and cardiac rehabilitation. We recruited a purposive sample of the following 3 stakeholder groups: (i) patients—adults (aged ≥ 18 years) discharged following a STEMI within the previous 3 months, who were able to provide informed consent and participate using English; (ii) healthcare providers—physicians, nurse practitioners, pharmacists, and allied health professionals directly involved in post-STEMI care; and (iii) health system leaders—administrative and clinical leaders with responsibility for planning and/or overseeing cardiology services in the region. Recruitment strategies included referrals from care teams, e-mail invitations, and snowball sampling.

### Data collection

Semi-structured interview guides were informed by the TDF and CFIR and tailored for each stakeholder group (only TDF for patients; combination of TDF and CFIR for healthcare providers and system leaders), with overlapping domains to facilitate comparison. Questions explored participants’ knowledge and beliefs about secondary prevention, their experiences with follow-up care, the availability of organizational supports, and the broader system-level barriers and enablers (see Supplemental Appendix S1 for the interview guide). Interviews were conducted virtually by a trained qualitative researcher (J.C.) between February 2023 and March 2024. Each interview lasted 30-60 minutes, was audio-recorded with verbal participant consent, and was transcribed verbatim. Transcripts were deidentified and imported into NVivo (QSR International, Denver, CO) for analysis. Data collection continued until “information power” was achieved, according to guidance by Malterud and colleagues.^29^

### Data analysis

We conducted a directed content analysis using TDF and CFIR. An initial coding framework was developed deductively from both frameworks while remaining open to inductive coding of novel concepts. Two research team members (J.C., M.C.) independently coded a subset of transcripts to refine the framework and ensure consistency. Discrepancies were resolved through discussion, with input from a senior investigator until consensus was achieved. The remaining transcripts were coded using the finalized framework. Coded data were synthesized within and across stakeholder groups to identify convergent and divergent barriers and enablers. Findings were discussed iteratively within the research team, emphasizing credibility, contextual variation, and alignment with study objectives. NVivo software (Lumivero, Denver, CO) facilitated coding and data management.

### Ethics

Ethical approval was obtained through the Hamilton Integrated Research Ethics Board (ID: 14624). All participants provided verbal informed consent prior to participation.

## Results

The study included 14 STEMI patients, 8 healthcare providers, and 4 system leaders (Table 1). Most of the patients were men (64%); the majority were experiencing their first STEMI (86%), and all reported having access to a primary care provider. Most had drug coverage (79%) and a caregiver at home (79%), and smoking rates were low (14%). Half of the patient sample was retired, with the remainder working or on disability. Healthcare providers represented a mix of acute academic (63%) and community hospital (37%) settings, with balanced gender representation. Our sample comprised 2 cardiologists, 3 general internists, 1 cardiac nurse, 1 hospital cardiac pharmacist, and 1 community pharmacist. Providers’ years of experience were evenly split, between 10-20 years and > 20 years. System leaders were evenly split by gender, and all had > 20 years of experience, reflecting their senior administrative and clinical leadership roles.

**Table 1:** Sample characteristics

Characteristic	Patients (n = 14)	Healthcare providers (n = 8)	Health system leaders (n = 4)
**Gender**			
Women	5 (36)	4 (50)	2 (50)
Men	9 (64)	4 (50)	2 (50)
**First STEMI**			
Yes	12 (86)	—	—
No	2 (14)	—	—
**Smoking status**			
Yes	2 (14)	—	—
No	12 (86)	—	—
**Drug coverage**			
Yes	11 (79)	—	—
No	3 (21)	—	—
**Primary care provider access**			
Yes	14 (100)	—	—
No	0 (0)	—	—
**Caregiver at home**			
Yes	11 (79)	—	—
No	3 (21)	—	—
**Working status**			
Working	6 (43)	—	—
Disabled	1 (7)	—	—
Retired	7 (50)	—	—
**Primary work site**			
Acute academic centre	—	5 (63)	—
Community hospital	—	3 (37)	—
**Years of healthcare experience**			
≥ 20	—	4 (50)	4 (100)
10–20	—	4 (50)	0 (0)

Values are n (%). STEMI, ST-elevation myocardial infarction.

### STEMI patient perspectives

STEMI patients described multiple influences on their recovery and follow-up care after STEMI. Six domains from the TDF were most prominent, as follows: knowledge; skills; beliefs about capabilities; behavioural regulation; social influences; and environmental context and resources.

A recurring theme was gaps in knowledge about symptoms, medications, and risk factors (TDF: knowledge domain). Some patients felt unprepared to manage their condition after discharge, underscoring the importance of clear and tailored communication. One patient highlighted how primary care helped bridge this gap:

“My family doctor explained my test results in detail and why it was so surprising that my cholesterol and blood pressure were normal, yet I still had a heart attack,” (Patient #10)

Patients also varied in their skills and confidence in managing medications and recognizing warning signs (TDF: skills domain). Structured guidance at discharge helped reinforce key messages; however, some patients still reported feeling underprepared, as evidenced by the following:

“When I got discharged, the nurse was very, very stern on the outcome of what happens if I do miss my medication, so that really stuck into my head. So I bring my medication with me wherever I go now,” (Patient #2)

Beliefs about recovery shaped patients’ willingness to engage with services such as cardiac rehabilitation (TDF: beliefs about capabilities domain). Although some expressed self-efficacy, others doubted their ability to sustain behavioural changes without structured support:

“I was hesitant to join cardiac rehab at first, but now I look forward to it because it shows me how much I can do.” (Patient #14)

Behavioural regulation strategies, including routines, reminders, and self-monitoring were key facilitators, although they required sustained effort and were vulnerable to disruption (TDF: behavioural regulation domain):

“I’ve always kept records, so I wanted him to keep a diary of information. Don’t write all the things, just write down what you’re taking, who you’re seeing, when you’re seeing them.” (Patient #1)

Social influences were consistently described as central to recovery. Family, peers, and coworkers offered practical help, encouragement, and accountability (TDF: social influences domain):

“My coworkers called regularly to check in, and it motivated me to get better and return to work.” (Patient #13)

Finally, patients emphasized barriers linked to the environmental context and resources, including transportation challenges, long distances to cardiac rehabilitation programs, and medication costs (TDF: environmental context and resources):

“I had to drive 40 minutes to attend rehab, which made it harder to commit weekly.” (Patient #12)

In summary, patient narratives highlight how knowledge, self-management skills, confidence, routines, social support, and access to resources influenced their ability to navigate the transition from hospital to community care, post-STEMI.

### Healthcare provider perspectives

Healthcare providers described their own challenges in supporting patient recovery and follow-up, with findings spanning both TDF domains and CFIR constructs.

A central concern was patient education and communication. Providers emphasized the difficulty of ensuring that patients understood and followed discharge instructions, particularly when language or literacy barriers were present (TDF: knowledge domain; CFIR: patient needs and resources construct):

“We have pamphlets, but if you can’t read it or you don’t understand it or don’t speak English it’s not helpful. I think they forget most of what we tell them.” (Provider #1)

Providers also described workflow gaps and lack of planning that led to fragmented coordination after discharge (CFIR: planning construct). Patients discharged late in the week or without clear follow-up instructions often became “lost in the system”:

“Patients fall through the cracks. They get discharged on a Friday night and by Monday nobody knows who’s following them.” (Provider #7)

Several providers identified opportunities to expand professional roles, especially for pharmacists and nurse practitioners, in enhancing medication management and adherence (TDF: social/professional role and identity domain; CFIR: networks and communications construct):

“Pharmacy has been left out, and they’re key. They know the meds, the interactions, the costs.” (Provider #2)

Psychological and emotional barriers were also highlighted. Providers recognized that fear, anxiety, or depression could prevent patients from engaging in rehabilitation or returning to regular physical activity (TDF: emotion domain; CFIR: patient needs and resources construct):

“Some are scared to exercise. They think they’ll bring on another heart attack. Or they’re depressed and don’t want to leave the house.” (Provider #4)

In addition, providers worried about the consequences of non-adherence, reinforcing the link between behaviours and outcomes (TDF: beliefs about consequences domain):

“We know if they don’t take their medication or come to rehabilitation they’ll be back in hospital.” (Provider #3)

From a CFIR perspective, providers discussed the complexity of regimens (CFIR: complexity construct), particularly when patients were prescribed multiple medications:

“Some patients take more than five medications daily, and it’s hard for them to keep track.” (Provider #1)

Views were mixed regarding innovations such as digital follow-up (CFIR: relative advantage construct). Although some providers recognized the potential benefits, others were skeptical:

“We’ve discussed digital health follow-ups, but some senior physicians don’t believe it will add much benefit.” (Provider #5)

Finally, providers highlighted the challenge of sustaining quality-improvement efforts within resource-constrained health systems (CFIR: readiness for implementation construct):

“Nobody has time to work on improvement initiatives because we’re working short, and nobody wants to come in on their own time.” (Provider #6)

Overall, providers underscored the interplay between patient-level challenges (education, motivation, adherence) and systemic barriers (resources, planning, coordination).

### Health system leader perspectives

Health system leaders discussed challenges and opportunities at organizational and structural levels, drawing attention to gaps in standardization, resources, and policy. Many highlighted inconsistencies in patient education and follow-up processes (CFIR: planning construct; TDF: knowledge domain). Each hospital or clinic developed its own approach, resulting in regional variability:

*“*Every hospital has their own way of doing things. There’s no standard patient education package, so it’s a bit hit or miss.” (Leader #1)

“There isn’t a clear pathway. Some patients get follow-up within a week, others not for months. It depends on where they live and who saw them in hospital.” (Leader #3)

Resource constraints were identified as a significant barrier to sustainability (CFIR: available resources construct). Leaders described reductions in rehabilitation programs and reliance on inconsistent funding sources:

“We’ve had to cut back our rehab service because of funding. We rely on short-term grants, but when those end, the programs shrink.” (Leader #3)

Implementation also was hampered by systemic factors such as bureaucratic delays and lack of ongoing financial commitment (CFIR: implementation climate construct; external policies and incentives construct):

“Good ideas always come up. We also do implementation projects, but then what happens is somehow it doesn’t translate to necessarily sustained, funded activity.” (Leader #4)

“We don’t have the funding to sustain innovations. The government expects us to implement new initiatives, but there’s no long-term financial commitment.” (Leader #2)

Despite these challenges, leaders noted promising adaptations. Virtual and hybrid follow-up models were seen as flexible ways to improve equitable access, especially for patients in rural areas (CFIR: adaptability construct):

“We set up a physician-led virtual call in the first month after discharge to assess medications, rehab attendance, and whether they have a specialist identified.” (Leader #4)

Finally, leaders observed that progress often relied on local champions (CFIR: engaging —champions construct). Sustained improvements were difficult without individual leaders pushing initiatives forward:

“It usually comes down to a few passionate people. If you don’t have a cardiologist or nurse championing it, it doesn’t happen.” (Leader #1)

### Summary across stakeholder groups

Across patients, healthcare providers, and system leaders, several areas of convergence were evident. All groups emphasized the central role of education and communication: patients often felt underprepared for recovery, healthcare providers struggled to reinforce information within limited time and workflows, and system leaders emphasized the lack of standardized approaches across institutions. Coordination of care was another universal concern; patients were uncertain about who was responsible for follow-up, healthcare providers described gaps that left individuals “falling through the cracks,” and system leaders noted the absence of consistent regional pathways. Resource constraints also were highlighted at every level; patients cited costs and travel, healthcare providers noted staff shortages and workload pressures, and system leaders described shrinking program capacity and reliance on unstable funding.

Important differences also emerged. Patients consistently underscored the value of social support networks, and healthcare providers focused on the potential of expanded roles for pharmacists and nurse practitioners to strengthen follow-up care. Health system leaders highlighted the importance of policy alignment and sustainable funding, with progress often dependent on local champions. Together, these findings illustrate how barriers to secondary prevention are interdependent and cascade across levels. But they also identified clear enablers, including family and peer support, interdisciplinary collaboration, and openness to virtual models of care. These insights informed a set of recommendations to strengthen post-STEMI follow-up care and secondary prevention, as summarized in Table 2.

**Table 2:** Set of recommendations to support the implementation of evidence-based secondary prevention for patients with ST-elevation myocardial infarction (STEMI)

Recommendation with relevant TDF and CFIR domains/constructs	Description
**Strengthen patient education and support systems** TDF domains: knowledge; beliefs about consequences CFIR construct: patient needs and resources	Implement structured, multi-touchpoint education on STEMI severity and secondary prevention, integrating pre-discharge counselling, follow-up calls, and pharmacist and/or primary care reinforcement. Standardize discharge counselling with input from physicians, nurses, and pharmacists, and leverage digital tools for ongoing education and adherence support.
**Improve interdisciplinary communication and care coordination** TDF domains: social/professional role and identity; environmental context and resources CFIR constructs: networks and communication; structural characteristics	Establishing standardized communication pathways among hospital specialists, pharmacists, primary care providers, and cardiac rehabilitation teams is essential for improving care transitions. Introducing standardized early post-discharge check-ins and expanding virtual follow-up options may help enhance accessibility, particularly for patients in rural areas.
**Address financial and structural barriers to care** TDF domains: environmental context and resources CFIR constructs: available resources; structural characteristics	Expand financial assistance programs, advocate for broader medication coverage, and develop hospital-based referral pathways to ensure all patients, including those without primary care providers, receive appropriate follow-up care.
**Implement structured follow-up and reminder systems** TDF domains: memory, attention, and decision processes CFIR construct: planning	Introduce automated follow-up systems (eg, text message reminders, patient-facing app-based communication and tracking) to reinforce medication adherence and appointment scheduling.
**Address psychological and behavioural barriers to adherence** TDF domains: beliefs about consequences; emotion CFIR construct: patient needs and resources	Integrate mental health support within cardiac rehabilitation programs, ensuring psychological counselling is available for patients experiencing anxiety, depression, or post-STEMI distress.
**Foster a supportive implementation climate for systemwide changes** TDF domains: environmental context and resources; social/professional role and identity CFIR constructs: readiness for implementation/Implementation climate; structural characteristics; external policy and incentives	Engage leadership and frontline staff in co-designing quality-improvement initiatives to reduce resistance to new processes and improve workforce retention. Advocate for sustainable funding models for secondary prevention initiatives, including dedicated resources for post-STEMI follow-up programs. Improve data-sharing infrastructure to allow seamless access to patient records for acute academic centres, community hospitals, and primary care providers.
**Expand and integrate cardiac rehabilitation services** TDF domains: social influences; environmental context and resources CFIR constructs: available resources; external policy and incentives	Cardiac rehabilitation should be integrated as a standard, opt-out service with pre-booked appointments to ensure all eligible patients receive care. Securing sustained funding is essential to expand capacity, reduce wait times, and facilitate early initiation post-discharge. Additionally, piloting hybrid models that combine virtual and in-person options can improve accessibility and participation, particularly for patients facing logistical barriers.
**Enhance pharmacist involvement in medication adherence support** TDF domains: skills; behavioural regulation CFIR construct: available resources	Establish structured pharmacist-led medication counselling before discharge and at key follow-up points. Encourage collaboration between hospital and community pharmacists to provide consistent post-STEMI medication education. Implement community pharmacist follow-ups to reinforce medication adherence and address concerns about side effects.

CFIR, Consolidated Framework for Implementation Research; TDF, Theoretical Domains Framework.

## Discussion

This multi-stakeholder, theory-informed qualitative study identified patient-, provider-, and system-level barriers and enablers to optimizing post-STEMI care transitions within a regional cardiac network in Ontario, Canada. Patients reported knowledge gaps, difficulties sustaining long-term routines, and logistical barriers to follow-up care. Providers and leaders emphasized fragmented communication, limited follow-up planning, and the absence of funding models to support ongoing implementation. Enablers included strong social support networks, expanded roles for nurse practitioners and pharmacists, and growing acceptance of virtual and hybrid models of care. Collectively, these findings underscore the need for coordinated and patient-centred strategies to strengthen secondary prevention following STEMI.^14^^,^^19^^,^^22^

Our findings reinforce prior research showing that patient education and postdischarge support are critical during recovery in promoting adherence and recovery following acute cardiac events.^5^^,^^6^ Consistent with previous Canadian and international studies, long-term adherence to secondary prevention therapies and participation in rehabilitation programs remain suboptimal, often linked to inadequate discharge planning and lack of follow-up continuity.^11^^,^^17^ Echoing findings from broader cardiovascular and chronic disease populations, our study demonstrates the impact of fragmented communication along with organizational and funding silos that impede sustained care coordination.^13^^,^^15^ However, important contextual differences exist across Canada. Some academic centres embed specialized cardiac pharmacists, streamline medication authorization processes, and operate province-wide rehabilitation networks (eg, Atlantic Cardiac Rehab Network), whereas rural and remote communities experience limited rehabilitation capacity and unreliable digital infrastructure.^12^^,^^22^^,^^23^ These differences mean that although the themes identified here are widely recognized, their impact and potential solutions vary across jurisdictions.

Our results point to several actionable strategies relevant to practice and policy. At the patient level, implementing structured, standardized discharge education could reduce knowledge gaps and enhance self-management confidence. At the provider level, formalizing interdisciplinary collaboration, including expanding the roles of nurse practitioners and pharmacists,^24^ could help strengthen continuity and reduce fragmentation. At the system level, developing standardized follow-up protocols, investing in digital infrastructure to support virtual care, and establishing sustainable funding models are essential for long-term implementation. Collectively, these strategies align with the recommendations derived from our data and summarized in Table 2. Existing programs in Ontario, such as Ontario Health at Home and the Integrated Heart Failure Care pathway,^30^ demonstrate viable models for coordinated, integrated follow-up care that could be adapted to post-STEMI with the right support, funding, and evaluation plan in place.

Key strengths of this study include the inclusion of patients, healthcare providers, and system leaders, allowing for examination of interconnected barriers and enablers across stakeholders at different levels of the healthcare framework. The use of established frameworks (TDF and CFIR) provides a systematic approach to identifying behavioural, organizational, and structural influences,^26^, ^27^, ^28^ which can be formally mapped onto intervention strategies. Moreover, this study highlights the fact that rapid, low-cost qualitative assessments can provide actionable insights for health-system improvement. The approach used here is highly replicable and could support other regions in identifying local barriers, highlighting enablers, and sharing best practices to improve follow-up care.

Although this analysis provides important insights, the findings are inherently context-specific. Regional variation in staffing models, rehabilitation availability, digital infrastructure, and funding arrangements may shape both the challenges observed and the feasibility of potential solutions. Given this possibility, transferability beyond this regional cardiac network should be interpreted with caution. The study’s modest sample size, particularly within the provider and leader groups, may further limit the breadth of experiences captured, although this approach remains appropriate for qualitative work aimed at thematic depth rather than representativeness. Nonetheless, many issues raised here align with patterns observed in broader cardiovascular and chronic disease literature, supporting their relevance beyond a single health region.

Future work should assess the effectiveness of the strategies outlined in Table 2. Priorities include evaluations of structured education programs, interdisciplinary follow-up models incorporating nonphysician providers, and virtual or hybrid care pathways. Replication of this study across multiple regions, including rural and urban settings, is also a clear opportunity to explore how contextual factors such as staffing models, virtual-care readiness, and rehabilitation capacity influence care transitions following STEMI. A coordinated provincial or national research initiative could support cross-site learning and help identify where enablers successfully implemented in some regions could be adapted elsewhere. Insights from this study have already informed the design of 2 patient-centred programs—a virtual STEMI follow-up clinic model^12^ and a comprehensive secondary prevention pathway for patients with atherosclerotic cardiovascular disease, led by the Canadian Cardiovascular Society.^19^ Plans to evaluate and implement programs such as these are ongoing (the secondary prevention pathway is being scoped for national scale-up), and a clear need is to link the effectiveness of these types of programs to key outcomes, including medication adherence, cardiac rehabilitation uptake, health service utilization, and long-term cardiovascular outcomes.

## Conclusion

Transitions in care following STEMI remain vulnerable to fragmentation, with downstream effects on adherence and participation in rehabilitation. This study identified multilevel barriers, including knowledge gaps, behavioural challenges, logistical constraints, suboptimal communication, and resource limitations, alongside important enablers, such as strong social support, interdisciplinary collaboration, and openness to virtual and/or hybrid models of care. By translating these insights into actionable recommendations, our study provides a practical roadmap for improving continuity and outcomes. Ensuring alignment among patient needs, provider capacity, and system-level supports is essential to effectively scaling and sustaining guideline-directed secondary prevention.
