# Supplementary material for: Strengthening Transitions in Care for Patients with ST-Elevation Myocardial Infarction: A Theory-Based Qualitative Study
Source: CJC Open. 2025 Dec 23;8(4):430–7. doi: 10.1016/j.cjco.2025.12.004 (PMC13084242; doi:10.1016/j.cjco.2025.12.004)
Supplement: Supplementary Material [file mmc1.pdf]

Semi-structured interview guide

Setting the scene:

- We want to speak to people with knowledge about the care processes/pathways involved in the care and support of patients with STEMI in our health region (e.g., HCPs, those in leadership roles, and patients). This will help us identify where the key barriers/gaps in care are and how this impacts how patients recover post-STEMI
- This interview guide includes open-ended questions exploring a broad set of factors influencing the implementation of evidence-based secondary prevention and follow-up post-STEMI in our health region (e.g., having access to and engaging with HCPs, lifestyle changes, medication adherence, access to and engaging with cardiac rehabilitation)

**Framework**	**Domain:** ***subdomain***	**Healthcare providers**	**Health system leaders**	**Patients with STEMI**
Theoretical Domains Framework (TDF)	Knowledge	Can you tell me what you know about secondary prevention and follow-up care post-STEMI<br><br>How familiar are you with this care process/pathway? / What do you know about this already?<br><br>Are there any gaps in what you know about it?	At the system level, can you tell me what you know about secondary prevention and follow-up care post-STEMI<br><br>How familiar are you with this care process/pathway? / What do you know about this already?<br><br>Are there any gaps in what you know about it?	Can you tell me what you know about the care process/pathway post-STEMI<br><br>How familiar are you with this care process/pathway? / What do you know about this already?<br><br>Are there any gaps in what you know about it?
	Skills	How easy or difficult would you find acting on what you know about secondary prevention and follow-up care post-STEMI	At the system level, do you think there are any particular skills required/involved in supporting secondary	Do you think there are any particular skills required/involved in navigating the care process/pathway post-STEMI

**Framework**	**Domain: *subdomain***	**Healthcare providers**	**Health system leaders**	**Patients with STEMI**
		Do you think there are any particular skills required/involved in supporting this care process/pathway?<br><br>Do you have the skills to support this care process/pathway?	prevention and follow-up care post-STEMI?<br><br>At the system level, do you have the skills to support this care process/pathway?	Do you have the skills to navigate the care process/pathway post-STEMI?
	Social/professional role and identity	What is your role in secondary prevention and follow-up care post-STEMI? And the role of others?<br><br>To what extent is supporting this care process/pathway part of your professional role?<br><br>Is it your job to do this?	At the system level, what is your role in secondary prevention and follow-up care post-STEMI? And the role of others?<br><br>To what extent is supporting this care process/pathway part of your professional role?<br><br>Is it your job to do this?	What is your role in navigating the care process/pathway post-STEMI? And the role of others?
	Beliefs about capabilities	How confident are you that you can support secondary prevention and follow-up care post-STEMI?<br><br>How well equipped are you to do this?	At the system level, how confident are you that patients (and healthcare providers) can be supported in secondary prevention and follow-up care post-STEMI?<br><br>How well equipped are you to do this?	How confident are you that you can navigate the care process/pathway post-STEMI?<br><br>How well equipped are you to do this?

**Framework**	**Domain: *subdomain***	**Healthcare providers**	**Health system leaders**	**Patients with STEMI**
	Optimism	How optimistic/pessimistic are you that supporting secondary prevention and follow-up care post-STEMI can improve patient care in the future?	At the system level, how optimistic/pessimistic are you that supporting secondary prevention and follow-up care post-STEMI can improve patient care in the future?	How optimistic/pessimistic are you about navigating the care process/pathway post-STEMI in the future?
	Beliefs about consequences	What do you think are the benefits of supporting secondary prevention and follow-up care post-STEMI?<br><br>What do you think are the costs of supporting secondary prevention and follow-up care post-STEMI?<br><br>In your opinion, do the benefits you have mentioned outweigh the costs?	At the system level, what do you think are the benefits of supporting secondary prevention and follow-up care post-STEMI?<br><br>At the system level, what do you think are the costs of supporting secondary prevention and follow-up care post-STEMI?<br><br>In your opinion, do the system-level benefits you have mentioned outweigh the system-level costs?	What do you think are the benefits of the care process/pathway post-STEMI as you know it?<br><br>What do you think are the costs of the care process/pathway post-STEMI as you know it?<br><br>In your opinion, do the benefits you have mentioned outweigh the costs?
	Reinforcement	To what extent do you received financial reimbursement to support secondary prevention and follow-up care post-STEMI?<br><br>To what extent do you received professional	At the system level, to what extent does financial reimbursement impact secondary prevention and follow-up care post-STEMI?<br><br>At the system level, to what extent does professional recognition impact	In what ways would successfully navigating the care process/pathway post-STEMI be rewarding?

Framework	Domain: *subdomain*	Healthcare providers	Health system leaders	Patients with STEMI
		recognition to support secondary prevention and follow-up care post-STEMI?	secondary prevention and follow-up care post-STEMI?	
	Intentions	How strong is your intention to support secondary prevention and follow-up care post-STEMI?	At the system level, how strong is your intention to support secondary prevention and follow-up care post-STEMI?	How strong is your intention to successfully navigate the care process/pathway post-STEMI?<br><br>What are some of the potential barriers to this?
	Goals	How much do you want to support secondary prevention and follow-up care post-STEMI?<br><br>Do you have any specific goals for supporting secondary prevention and follow-up care post-STEMI?<br><br>What are the incentives for supporting this care process/pathway?	At the system level, how much do you want to support secondary prevention and follow-up care post-STEMI?<br><br>At the system level, are there any specific goals for supporting secondary prevention and follow-up care post-STEMI?<br><br>At the system level, what are the incentives for supporting this care process/pathway?	How much of a priority is successfully navigating the care process/pathway post-STEMI? What might be competing priorities?<br>Do you have any specific goals for your post-STEMI care?<br><br>What are the incentives for successfully navigating the care process/pathway post-STEMI?
	Memory, attention, and decision processes	Is following the guidance around secondary prevention and follow-up	At the system level, is following the guidance around secondary	Is navigating the care process/pathway post-STEMI something you usually do?

Framework	Domain: ***subdomain***	Healthcare providers	Health system leaders	Patients with STEMI
		care post-STEMI something you usually do?<br><br>Will you remember to do this in future?	prevention and follow-up care post-STEMI something that is usually done?	Will you remember to do this in the future?
	Environment context and resources	What environmental factors or resources help or hinder supporting secondary prevention and follow-up care post-STEMI?<br><br>Do the systems in place help or hinder your ability to do this?	At the system level, what environmental factors or resources help or hinder supporting secondary prevention and follow-up care post-STEMI?	What environmental factors or resources help or hinder you navigating the care process/pathway post-STEMI?<br><br>Do the systems in place help or hinder your ability to do this?
	Social influences	Do people you work with support secondary prevention and follow-up care post-STEMI?<br><br>Do others you work with support you to do this?<br><br>Do you feel under pressure from anyone to do this? Or not to do it?	At the system level, do people you work with support secondary prevention and follow-up care post-STEMI?<br><br>At the system level, do you feel under pressure from anyone to do this? Or not to do it?	Do you have people who support you to navigate the care process/pathway post-STEMI?<br><br>Do you feel under pressure from anyone to do this? Or not to do it?
	Emotion	To what extent does the psychological working environment (e.g., stress) affect how you support secondary prevention and	At the system level, to what extent does the psychological working environment (e.g., stress) affect secondary prevention	To what extent does emotion and/or stress affect how you navigate the care process/pathway post-STEMI?

Framework	Domain: ***subdomain***	Healthcare providers	Health system leaders	Patients with STEMI
		follow-up care post-STEMI?	and follow-up care post-STEMI?	What emotions come to mind when you think about your STEMI and your care?
	Behavioural regulation	Are there things you need to do before you can support secondary prevention and follow-up care post-STEMI?<br><br>Are there things that help to prompt you to support this care process/pathway?<br><br>Are there particular types of patients for whom supporting this care process/pathway is more difficult?<br><br>Do you ever receive feedback on your performance in supporting secondary prevention and follow-up care post-STEMI?	At the system level, are there things you need to do before you can support secondary prevention and follow-up care post-STEMI?<br><br>At the system level, are there things that help to prompt you to support this care process/pathway?<br><br>Are there particular types of patients for whom supporting this care process/pathway is more difficult?<br><br>At the system level, do you ever receive feedback on performance in supporting secondary prevention and follow-up care post-STEMI?	Are there things you need to do before you can navigate the care process/pathway post-STEMI?<br><br>Are there things that help to prompt you to navigate the care process/pathway post-STEMI?<br><br>Are there things that help you monitor as you go along the care process/pathway post-STEMI?
	Any other business	Are there any other factors that you think might be important that we haven't covered?	Are there any other factors that you think might be important that we haven't covered?	Are there any other factors that you think might be important that we haven't covered?

**Framework**	**Domain: *subdomain***	**Healthcare providers**	**Health system leaders**	**Patients with STEMI**
Consolidated Framework for Implementation Research (CFIR)	Intervention characteristics: *Evidence Strength & Quality*	What do influential stakeholders or leaders think of evidence-based secondary prevention and follow-up post-STEMI?<br><br>What kind of supporting evidence or proof is needed about the effectiveness of this care process/pathway to get staff (or others) on board?	What do other influential stakeholders or leaders think of evidence-based secondary prevention and follow-up post-STEMI?<br><br>What kind of supporting evidence or proof is needed about the effectiveness of this care process/pathway to get staff (or others) on board?	**Given CFIR focuses on implementation at the HCP/organization/ system level, this section is N/A for patients.**
	Intervention characteristics: *Adaptability*	What kinds of changes or alterations do you think you will need to make to this care process/pathway so it will work effectively in your organization?	What kinds of changes or alterations do you think you will need to make to this care process/pathway so it will work effectively in your organization/system?	
	Intervention characteristics: *Complexity*	How complicated is supporting evidence-based secondary prevention and follow-up post-STEMI? And how would this impact implementation?	How complicated is supporting evidence-based secondary prevention and follow-up post-STEMI? And how would this impact implementation?	
	Intervention characteristics: *Cost*	What costs will be incurred to implement evidence-based secondary prevention and follow-up post-STEMI?	What costs will be incurred to implement evidence-based secondary prevention and follow-up post-STEMI?	

**Framework**	**Domain: *subdomain***	**Healthcare providers**	**Health system leaders**	**Patients with STEMI**
	Outer setting: *Patient Needs & Resources*	To what extent are the needs and preferences of the individuals served by your organization considered when implementing changes in care processes/pathways?<br><br>How well do you think evidence-based secondary prevention and follow-up post-STEMI will meet the needs of the individuals served by your organization?	To what extent are the needs and preferences of the individuals served by your organization/system considered when implementing changes in care processes/pathways?<br><br>How well do you think evidence-based secondary prevention and follow-up post-STEMI will meet the needs of the individuals served by your organization/system?	
	Outer setting: *Peer pressure*	Can you tell me what you know about any other organizations that have implemented evidence-based secondary prevention and follow-up post-STEMI or other similar care processes/pathways?	Can you tell me what you know about any other organizations/systems that have implemented evidence-based secondary prevention and follow-up post-STEMI or other similar care processes/pathways?	
	Outer setting: *External Policies & Incentives*	What kind of local, state, or national performance measures, policies, regulations, or guidelines might influence the implementation of evidence-based secondary	What kind of local, state, or national performance measures, policies, regulations, or guidelines might influence the implementation of evidence-based secondary	

Framework	Domain: ***subdomain***	Healthcare providers	Health system leaders	Patients with STEMI
		prevention and follow-up post-STEMI?	prevention and follow-up post-STEMI?	
	Inner setting: *Structural Characteristics*	How will the infrastructure of your organization (social architecture, age, maturity, size, or physical layout) affect the implementation of evidence-based secondary prevention and follow-up post-STEMI?<br><br>What kinds of infrastructure changes will be needed to accommodate the implementation of evidence-based secondary prevention and follow-up post-STEMI?	How will the infrastructure of your organization/system (social architecture, age, maturity, size, or physical layout) affect the implementation of evidence-based secondary prevention and follow-up post-STEMI?<br><br>What kinds of infrastructure changes will be needed to accommodate the implementation of evidence-based secondary prevention and follow-up post-STEMI?	
	Inner setting: *Networks & Communications*	Can you describe your working relationship with leaders?<br><br>Can you describe your working relationship with influential stakeholders?	Can you describe your working relationship with other leaders?<br><br>Can you describe your working relationship with other influential stakeholders?	
	Inner setting: *Culture*	How would you describe the culture of your organization?	How would you describe the culture of your organization/system?	

Framework	Domain: ***subdomain***	Healthcare providers	Health system leaders	Patients with STEMI
		To what extent are new ideas embraced and used to make improvements in your organization?	To what extent are new ideas embraced and used to make improvements in your organization/system?	
	Inner setting: *Implementation climate*	What is the general level of receptivity in your organization to implementing changes in care processes/pathways?	What is the general level of receptivity in your organization/system to implementing changes in care processes/pathways?	
	Inner setting: *Learning climate*	To what extent do you feel like you can try new things to improve your work/care processes/pathways?	To what extent do you feel like you can try new things to improve your work/care processes/pathways?	
	Inner setting: *Available resources*	Do you expect to have sufficient resources to implement evidence-based secondary prevention and follow-up post-STEMI?	Do you expect to have sufficient resources to implement evidence-based secondary prevention and follow-up post-STEMI?	
	Process: *Opinion leaders*	Who are the key influential individuals to get on board to help implement evidence-based secondary prevention and follow-up post-STEMI?	Who are the key influential individuals to get on board to help implement evidence-based secondary prevention and follow-up post-STEMI?	
	Process: *Reflecting & Evaluating*	What kind of information would you plan to collect as you implement evidence-based secondary	What kind of information would you plan to collect as you implement evidence-based secondary	

Framework	Domain: ***subdomain***	Healthcare providers	Health system leaders	Patients with STEMI
		prevention and follow-up post-STEMI?<br><br>To what extent would your organization set goals for implementing evidence-based secondary prevention and follow-up post-STEMI?<br><br>How would you assess progress towards goals related to evidence-based secondary prevention and follow-up post-STEMI?	prevention and follow-up post-STEMI?<br><br>To what extent would your organization/system set goals for implementing evidence-based secondary prevention and follow-up post-STEMI?<br><br>How would you assess progress towards goals related to evidence-based secondary prevention and follow-up post-STEMI?	
